# 肺腺癌脊柱定向转移的分子机制与精准诊疗研究进展

**DOI:** 10.3779/j.issn.1009-3419.2026.102.19

**Published:** 2026-06-20

**Authors:** Hongyang FU, Yitong SHE, Manglai LI, Yiming DU, Yong ZHU

**Affiliations:** ^1^010010 呼和浩特，内蒙古医科大学研究生院（付弘扬，折依侗，杜一鸣）; ^1^Inner Mongolia Medical University; ^2^北京大学肿瘤医院内蒙古医院（李芒来，祝勇）; ^2^Peking University Cancer Hospital Inner Mongolia Hospital, Hohhot 010010, China

**Keywords:** 肺肿瘤, 脊柱定向转移, 脊柱微环境, 精准诊疗, Lung neoplasms, Spinal organotropic metastasis, Spinal microenvironment, Precision therapy

## Abstract

肺腺癌（lung adenocarcinoma, LUAD）是非小细胞肺癌（non-small cell lung cancer, NSCLC）中最常见的亚型，脊柱转移发生率高且预后较差。LUAD的脊柱转移并非单纯的骨转移，而是由脊柱解剖特性、微环境特异性及分子调控机制共同介导的定向转移过程，但现有研究多聚焦于泛骨转移，针对脊柱定向转移的专属机制与精准诊疗体系尚未形成。因此，本文从LUAD脊柱定向转移潜能、微环境调控、转移途径3个关键方面，系统阐述其分子机制，并结合脊柱转移的临床特点与当前研究不足，为LUAD脊柱转移的基础研究和临床精准防治提供新思路。

## Abstract (en)

Lung adenocarcinoma (LUAD) is the most common subtype of non-small cell lung cancer (NSCLC), with a high incidence of spinal metastasis and poor prognosis. Spinal metastasis of LUAD is not merely a simple bone metastasis but a directed metastasis process mediated by the anatomical characteristics of the spine, microenvironment specificity, and molecular regulatory mechanisms. However, current research mainly focuses on general bone metastasis, and a dedicated mechanism and precise diagnosis and treatment system for spinal-directed metastasis have not yet been established. Therefore, this article systematically elaborates on the molecular mechanisms of LUAD spinal-directed metastasis from three key aspects: metastatic potential, microenvironment regulation, and metastasis pathways. Combined with the clinical characteristics of spinal metastasis and the current research deficiencies, it provides new ideas for basic research and clinical precise prevention and treatment of LUAD spinal metastasis.

非小细胞肺癌（non-small cell lung cancer, NSCLC）是全球最常见的恶性疾病类型之一，也是癌症相关死亡的主要原因^[1]^。其中肺腺癌（lung adenocarcinoma, LUAD）是NSCLC中脊柱转移发生率最高的亚型^[2]^。研究^[3]^表明LUAD是其最常见的病理类型，胸腰椎是脊柱转移的好发部位，并伴随疼痛、病理性骨折、脊髓压迫等并发症，整体预后较其他肿瘤来源的脊柱转移患者更差，这一特征与LUAD独特的生物学行为密切相关，也是临床诊疗中需重点关注的问题^[4,5]^。

目前研究多聚焦于肺癌骨转移，忽视了脊柱转移中解剖结构、局部微环境及分子调控层面的独特性。此外，临床诊疗亦多沿用泛骨转移策略，未充分考虑脊柱转移易发生脊髓压迫、药物渗透性差、局部治疗剂量受限等临床问题，诊疗精准性不足。因此，本文聚焦LUAD脊柱定向转移相关调控通路展开综述，为临床精准干预提供依据。

## 1 LUAD细胞脊柱定向转移的潜能与分子特征

LUAD细胞可通过驱动基因突变、激活上皮-间质转化（epithelial-mesenchymal transition, EMT）等多重调控途径形成协同作用网络，进而获得向脊柱定向转移的潜能。

LUAD在分子水平上表现出显著的异质性。有研究^[6]^通过对2532例LUAD样本的临床病理与基因组进行整合分析，证实发生转移的LUAD具有独特基因组特征，其中*TP53*、*SMARCA4*、*CDKN2A*失活突变可显著影响转移的发生时间与器官倾向性，揭示了肿瘤细胞的突变负荷可能在LUAD脊柱转移中起作用。并且已有多项研究^[7,8]^证实，表皮生长因子受体（epidermal growth factor receptor, *EGFR*）基因突变是LUAD中的关键驱动因素，其可通过调控核因子κB受体活化因子配体/骨保护素（receptor activator of nuclear factor-κB ligand/osteoprotegerin, RANKL/OPG）通路激活破骨细胞（osteoclast, OC），赋予肿瘤细胞定植与骨破坏特性。需注意，RANKL/OPG介导的OC活化通路广泛存在于各类溶骨性骨转移病灶，仅参与骨基质溶解，并不具备介导肿瘤特异性黏附椎体的作用。*EGFR*突变之所以能够增强LUAD对脊柱的趋向性，其中一条关键轴是LUAD细胞可特异性分泌人趋化因子配体17（CXC chemokine ligand 17, CXCL17），该因子经类固醇受体共激活因子/黏着斑激酶（steroid receptor coactivator/focal adhesion kinase, Src/FAK）信号介导肿瘤与椎体微环境特异性互作，是推动肿瘤脊柱定植的重要分子基础^[9]^。

除驱动基因突变赋予LUAD细胞内在侵袭与定向转移潜能外，EMT是LUAD细胞获得转移能力的关键生物学过程，二者共同构成发生转移的内在动力。EMT可使肿瘤细胞下调E-钙黏蛋白、上调N-钙黏蛋白及波形蛋白表达，进而丧失上皮细胞极性与细胞间黏附能力，转化为具有高迁移、高侵袭特性的间质表型，更易穿透基底膜与血管壁^[10]^。但EMT并非独立发挥作用，它可在相关脊柱特异性分子的调控下，进一步增强肿瘤细胞的转移潜能。Wang等^[11]^研究提到，hsa_circ_0006571通过“分子海绵”机制吸附miR-138，解除其对Sirt1的抑制，Sirt1上调后可激活EMT，增强LUAD细胞的脊柱转移能力。

因此综合来看，RANKL/OPG、EMT等分子通路是溶骨性骨转移共有的调控模式，CXCL17、hsa_circ_0006571是在脊柱转移中特异性富集的信号。二者分层协作，前者赋予肿瘤细胞脊柱靶向属性，后者提供椎体骨髓黏附条件，共同决定LUAD脊柱定向转移的器官倾向性。

## 2 脊柱解剖与血行转移途径

LUAD多起源于肺外周胸膜下区域，紧邻胸椎椎旁软组织，肿瘤进展突破胸膜后可直接侵袭椎旁间隙^[12,13]^。此外，人体血管系统的分布为肿瘤细胞的侵袭提供了动力，肿瘤细胞可侵入肺静脉系统，经肺静脉回流至左心房与左心室，再通过体循环动脉系统直接灌注至胸椎椎体^[14]^。胸椎的血供主要来源于肋间后动脉与腰动脉分支，属于体循环直接供血的核心区域，血供分布密集且灌注压力稳定。与四肢长骨相比，胸椎椎体内部血供更为丰富、血窦腔隙更宽大，血流速度相对缓慢，更有利于肿瘤细胞栓子滞留、黏附并穿出血管壁，在椎体内部形成早期转移灶。另外脊柱富含红骨髓，拥有密集且迂曲的血管网络，其中与肺静脉、肋间静脉广泛吻合交通的Batson椎静脉丛为无瓣膜结构的静脉网络，是LUAD胸椎转移的重要通道。当咳嗽、憋气、负重或体位剧烈变动引发胸腹腔内压力一过性升高时，该静脉丛内血液易发生逆向反流，可将肺部脱落的肿瘤细胞不经肺循环、肝脏滤过，直接快速输送至胸椎椎体内部的静脉窦内，实现低阻力、高效率的脊柱定向转移^[15,16]^。

依托上述解剖与肿瘤生物学优势，血源性播散成为LUAD脊柱转移的核心途径，具体过程可分为“侵袭-入血-滞留-定植”。首先，LUAD细胞经EMT及相关分子介导基质降解突破肺基底膜，侵入肺静脉或支气管动脉进入体循环；随后，部分LUAD细胞经Batson椎静脉丛逆行抵达脊柱，其余经动脉系统汇入脊柱血管床。由于脊柱血管流速缓、分支多，利于LUAD细胞黏附滞留于血管内皮，同时其分泌的血管通透因子可破坏内皮屏障，促使LUAD细胞迁出血管进入骨髓微环境；最终LUAD细胞依托脊柱微环境增殖，形成脊柱转移灶^[17,18]^。

## 3 脊柱微环境定植

### 3.1 外泌体调控转移前生态位

外泌体调控转移前生态位（pre-metastatic niche, PMN）为远处器官中的支持性微环境，通过免疫抑制、炎症信号传导、血管、淋巴管生成、促器官性以及代谢重编程等因素重塑原发肿瘤，以促进转移定植。其中外泌体作为介导细胞间远程信息交流的重要膜性囊泡载体，可重塑远处组织微环境并诱导PMN形成，进而主导肿瘤的器官靶向转移特性^[19]^。相关研究^[20]^表明，LUAD来源外泌体很可能作为允许因子，配合缺氧、细胞因子等局部微环境因素诱导PMN的构建，其可携带整合素、miRNA及蛋白等LUAD特异性分子信号，通过靶向特定器官细胞重塑微环境，促进LUAD细胞的远处转移。而这些活性物质经由体循环血流，借助Batson椎静脉丛的独特解剖通路可快速定植于脊柱。四肢骨因缺乏Batson椎静脉丛这一快速递送通道，肿瘤外泌体经动脉分散分布，难以高效富集并提前构建PMN。而脊柱可借助独特静脉通路快速富集肿瘤源性外泌体，外泌体被基质、免疫及破骨祖细胞摄取后，重塑局部免疫、血管与骨代谢稳态并激活EMT，提前搭建利于循环肿瘤细胞（circulating tumor cells, CTCs）黏附增殖的微环境，大幅提升脊柱对LUAD细胞的转移适配性^[21,22]^。

### 3.2 脊柱细胞与分子微环境

脊柱骨组织主要由成骨细胞（osteoblast, OB）、OC及骨髓基质细胞共同构成，各类细胞通过分泌多种活性因子形成复杂的调控网络^[4,17]^。研究^[23]^提到，OC进行骨吸收时会释放转化生长因子-β（transforming growth factor-β, TGF-β）、血小板衍生生长因子（platelet-derived growth factor, PDGF）等基质储存因子；同时，骨质破坏还能诱导肿瘤分泌甲状旁腺素相关肽（parathyroid hormone related peptide, PTHrP）及肿瘤坏死因子-α（tumor necrosis factor-α, TNF-α）、白细胞介素-1（interleukin-1, IL-1）等炎症因子，上调RANKL表达并持续激活OC，形成促LUAD增殖转移的恶性循环。上述骨代谢调控模式广泛存在于全身溶骨性骨转移病灶中，由此可推断该通路同样参与脊柱转移灶的进展过程。与此同时，唾液酸免疫球蛋白型凝集素-15（sialic acid-binding immunoglobulin-type lectin-15, Siglec-15）在LUAD脊柱转移灶中的表达显著高于原发灶及癌旁组织，其既可抑制肿瘤微环境（tumor microenvironment, TME）中的抗肿瘤T细胞反应，又能驱动OC分化及骨吸收，与LUAD脊柱转移密切相关^[24,25]^。

另外，骨髓基质细胞的作用也尤为关键。一方面，基质细胞可通过旁分泌脂肪因子、炎症因子维持OB、OC功能平衡与局部免疫稳态；另一方面，微环境信号级联可诱导肿瘤细胞发生EMT，重塑细胞表型以提升整体转移能力^[26]^。其中，脊柱骨髓内皮细胞特异性分泌C-X3-C基序趋化因子配体1（chemokine C-X3-C ligand 1, CX3CL1），一方面通过CX3CL1/C-X3-C趋化因子受体1（C-X3-C chemokine receptor 1, CX3CR1）通路上调细胞间黏附因子-1（intercellular adhesion molecule-1, ICAM-1），结合肿瘤细胞表面淋巴细胞功能相关抗原-1（leukocyte function-associated antigen 1, LFA-1），介导CTCs黏附；另一方面激活Src/鸟苷酸交换因子H1（guanine nucleotide exchange factor H1, GEF-H1）通路提升血管通透性，促进肿瘤细胞跨内皮迁移，特异性驱动LUAD脊柱转移^[27]^。此外，定植椎体的LUAD细胞可分泌CXCL17，激活Src/FAK通路，招募单核巨噬细胞至红骨髓分化为促瘤M2巨噬细胞，进而加速转移灶增殖^[9]^。

综上，脊柱独特解剖结构、血行通路、外泌体预调控及局部微环境共同构成LUAD转移的适宜条件，契合肿瘤“种子-土壤”理论（图1）。肿瘤细胞作为转移种子，依靠脊柱微环境这一特异性土壤完成定植增殖，二者特异性相互作用是LUAD易发生脊柱转移的重要因素^[28]^。

**图 1:**
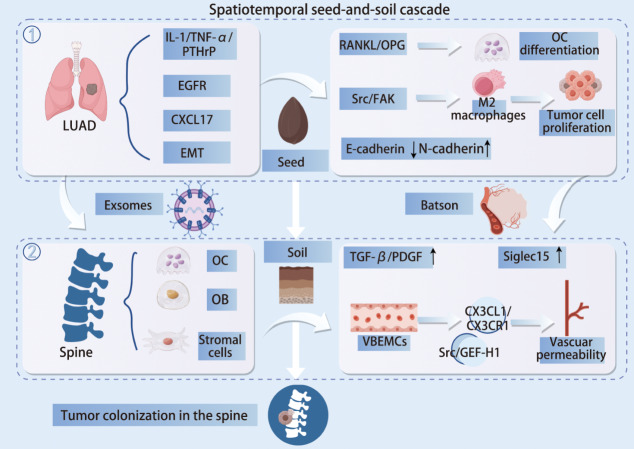
种子-土壤时空过程。LUAD作为“种子”，经多分子通路与外泌体调控脊柱微环境“土壤”，依靠Batson静脉通路实现脊柱转移定植。

## 4 LUAD脊柱转移的精准诊疗

### 4.1 早期诊断

现有诊断手段多基于泛骨转移，缺乏脊柱特异性，导致微转移检出率低、精准性不足。因此，构建“影像学分层+脊柱专属标志物联合+风险预测模型”的三维早期诊断体系，有助于实现脊柱微转移早发现和高危人群的精准识别。

#### 4.1.1 影像学分层

影像学检查是LUAD脊柱转移早期诊断的核心手段，临床应建立脊柱专属的影像学分层诊断流程，替代泛骨转移通用检查方案。磁共振成像（magnetic resonance imaging, MRI）可通过多序列成像清晰界定病灶范围，并且可用于发射计算机断层扫描技术（emission computed tomography, ECT）或计算机断层扫描（computed tomography, CT）对可疑病灶的定性验证，是脊柱转移诊断的金标准^[29]^。与此同时，正电子发射断层扫描/CT（positron emission tomography/CT, PET/CT）技术能够有效整合代谢活性与解剖结构信息，不仅可用于*EGFR*突变、CXCL17高表达等LUAD脊柱转移高危人群的全身评估，还可反映肿瘤经Batson椎静脉丛播散的全身分布特征，从而辅助判断转移范围。相关小鼠实验^[30]^证实其诊断敏感性达92.19%，特异性为78.95%，显著优于MRI。临床案例^[31]^同样显示其可在发现肺原发灶的同时检查脊柱的转移灶，并且结合MRI验证能进一步提升诊断准确率。在此基础上，PET/CT与MRI多模态影像融合可互补解剖定位与代谢评估优势，其中增强MRI联合PET融合成像诊断效能最佳，灵敏度100%、特异度92%、阴性预测值100%，能有效降低漏诊率^[32]^。此外，基于该融合技术的导航辅助活检，通过导入多模态数据、标记肿瘤范围、以PET高代谢SUVmax位点为核心靶点，术中实时匹配影像、精准规划穿刺路径，适用于微小、解剖复杂及仅MRI显影的病灶^[33]^。因此，“MRI+PET/CT”联合诊断模式能够充分发挥两种影像的互补价值，显著提升LUAD脊柱微转移的检出率与诊断准确性，在临床的早期评估中具有重要诊断价值。

#### 4.1.2 标志物联合检测

转移相关标志物检测是LUAD脊柱转移早期诊断的重要补充，可弥补影像难以识别早期脊柱微转移的短板。针对传统标志物缺乏脊柱特异性、难以区分脊柱转移与其他骨病变的问题，可构建“脊柱特异性分子标志物+骨代谢标志物+LUAD核心标志物”三联检测方案，提升早期诊断灵敏度与特异度。其中，蛋白质类标志物CXCL17、Siglec15等，经研究^[9,25]^证实具有明确的脊柱转移特异性，可作为脊柱专属分子标志物的核心组分。同时，非编码RNA（noncoding RNA, ncRNA）、CTCs与循环肿瘤DNA（circulating tumor DNA, ctDNA）作为液体活检的重要组成部分，可动态反映肿瘤负荷与基因突变状态，有助于早期发现LUAD细胞微转移及评估治疗反应^[34-36]^。此外，I型胶原羧基末端肽（C-terminal telopeptide of type I collagen, CTX）、骨特异性碱性磷酸酶（bone-specific alkaline phosphatase, BALP）等作为骨代谢标志物在溶骨性转移早期即可出现明显升高，提示骨破坏现象^[4,37]^。而LUAD的核心标志物[癌胚抗原（carcinoembryonic antigen, CEA）与细胞角蛋白19片段（cytokeratin 19 fragment, CYFRA21-1）]可明确肿瘤来源，与脊柱专属标志物、骨代谢标志物联合使用可显著提升诊断特异性^[38]^。

但目前三联标志物联合检测缺乏充足的临床证据，难以推广；且存在外周血靶标浓度低、检测标准不一、良性骨病干扰、检测成本高等转化难题。后续需先经单中心研究明确诊断临界值，再通过多中心前瞻性队列验证效能，同步优化检测技术、统一判定阈值、降低成本，推动方案标准化落地。

#### 4.1.3 风险预测模型构建

基于临床病理特征、分子标志物与影像学参数构建LUAD脊柱转移专属风险预测模型，可显著提升早期筛查精准度，为临床分层管理提供科学依据。

在模型构建方面，已有多项研究为脊柱转移专属预测模型提供了扎实的方法学基础。Qi等^[39]^研究纳入511例肺癌患者，结合临床特征与多项循环肿瘤标志物构建肺癌转移列线图，整体预测效能稳定。该模型操作简便易解读，适合基层临床快速风险初筛，但维度单一，对脊柱微转移预测精度不足，仍需大样本多中心队列进一步验证。在此基础上，Jiang等^[40]^研究进一步聚焦LUAD腰椎脊柱转移人群，依托3.0T多参数MRI影像组学与深度学习构建融合模型，无创术前评估*EGFR*突变，训练集曲线下面积（area under the curve, AUC）为0.891，验证集AUC为0.771，灵敏度与特异性均表现优异。但模型依赖高端影像设备，数据预处理复杂、可移植性较弱，普及应用受限。

相较而言，Zhang等^[41]^基于监测、流行病学和最终结果（The Surveillance, Epidemiology, and End Results, SEER）数据库的大规模LUAD队列，对比7种机器学习算法后证实XGBoost模型在转移预测中性能最优，准确率与泛化能力突出，且配套了在线工具便于临床使用。整体来看，传统统计模型易解读但精度有限，深度学习模型效能高却通用性不足，XGBoost等集成机器学习模型可作为当前LUAD脊柱转移风险预测的临床优选模型。与此同时，人工智能（artificial intelligence, AI）技术在脊柱转移灶的节段特异性检测中展现出独特优势，可作为风险预测模型的重要补充手段^[42]^，有效弥补单一模型的短板。

因此，临床中可将LUAD脊柱转移专属风险预测模型与AI影像节段特异性检测技术有机整合，形成“先精准筛高危、再定位识病灶”的闭环诊疗流程，实现LUAD脊柱转移的精准预测、分层管理与早期防控，切实改善患者预后。

### 4.2 精准治疗

针对LUAD脊柱转移的治疗，应围绕预防转移、控制进展、缓解症状、逆转耐药四大核心方向，结合脊柱转移的特异性，构建分型预防+分层治疗+耐药逆转的一体化精准治疗体系。其中分型预防是从源头抑制脊柱转移潜能的关键，需聚焦脊柱定向转移的相关分子特征，而非单纯依赖驱动基因分型，例如*EGFR*突变+CXCL17高表达型、Siglec15高表达型等，通过精准靶向脊柱转移特异性分子通路实现早期干预。目前此类针对性研究仍较为匮乏，亟待深入探索，例如Siglec15抗体联合骨靶向修饰药物是否可实现LUAD脊柱转移的精准治疗，值得进一步验证。

在靶向治疗方面，第三代EGFR-酪氨酸激酶抑制剂（EGFR-tyrosine kinase inhibitors, EGFR-TKIs）能有效抑制*EGFR*突变LUAD侵袭转移，尤其适合术后高危患者预防转移。当耐药合并V-raf鼠肉瘤病毒癌基因同源体B1（V-raf murine sarcoma viral oncogene homolog B1, *BRAF*） V600E突变，采用达拉非尼+曲美替尼+奥希替尼的EGFR/BRAF/MEK三重联合靶向方案可阻断转移通路，客观缓解率为61.5%，疾病控制率为92.3%，中位无进展生存期（progression-free survival, PFS）延长至13.5个月，可作为LUAD脊柱转移靶向治疗方案^[43,44]^。

此外，免疫检查点阻断（immune checkpoint blockade, ICB）是LUAD重要的全身治疗手段，该疗法能够阻断程序性细胞死亡配体1（programmed cell death ligand 1, PD-L1）/程序性细胞死亡受体1（programmed cell death-1, PD-1）抑制通路，激活TME内耗竭的T细胞，重塑全身抗肿瘤免疫以抑制远处转移^[45]^。但ICB耐药仍是临床关键难题，其核心原因包括脊柱转移微环境高度免疫抑制、Batson椎静脉丛药物通透性差等。针对上述耐药问题，可通过优化药物递送系统提高脊柱病灶药物浓度。具体而言，外泌体具备优良的生物相容性，可装载化疗药物、核酸片段，借助电穿孔技术完成药物加载，再经靶向修饰后定向作用于病灶。已有实验^[46,47]^证实，嵌合抗原受体T细胞（chimeric antigen receptor T cell, CAR-T）改造得到的工程外泌体可有效缩减肿瘤病灶、延长荷瘤小鼠生存时间。同时，Liang等^[24]^针对脊柱转移高表达的Siglec15靶点，构建谷胱甘肽响应骨靶向MnO₂纳米载体，依托Asp⁸多肽富集于脊柱转移灶，共载Siglec15 siRNA与顺铂。该体系可沉默Siglec15以解除免疫抑制、激活CD8^+ ^T细胞，同时抑制OC活化、阻断骨溶解，兼具免疫化疗抑瘤与骨保护作用，为LUAD脊柱转移提供了新型高效治疗策略。

而对于已发生脊柱转移的患者，早期局部干预是控制症状、避免神经功能损伤的核心。立体定向放疗（stereotactic body radiation therapy, SBRT）凭借精准靶向优势，可高效杀伤转移灶、快速缓解疼痛。在脊柱转移专属放疗方案中，胸腰椎转移可采用较高剂量SBRT；颈椎转移因紧邻脊髓，需采用低剂量分次放疗以降低神经损伤风险。SBRT可将高剂量辐射精准送达骨转移部位，实现持久肿瘤控制与症状缓解，为局部治疗提供可靠支撑^[48,49]^。

综上，现阶段各类肿瘤精准诊疗技术分属基础研究、临床试验、临床应用三大发展阶段。其中影像分层、EGFR靶向药物、免疫治疗以及脊柱SBRT已投入常规临床使用，不过免疫治疗会受椎体免疫抑制微环境与椎静脉给药屏障影响，临床转化仍存在明显阻碍；脊柱专属三联标志物仅完成小样本临床探索；各类AI预测工具、骨靶向纳米和外泌体递送载体仅停留在细胞动物实验阶段，尚未开展人体研究。后续需清晰各类成果所处研发阶段，结合LUAD脊柱转移的专属特点搭建一体化全流程诊疗体系，全面提升诊疗水平（图2）。

**图 2:**
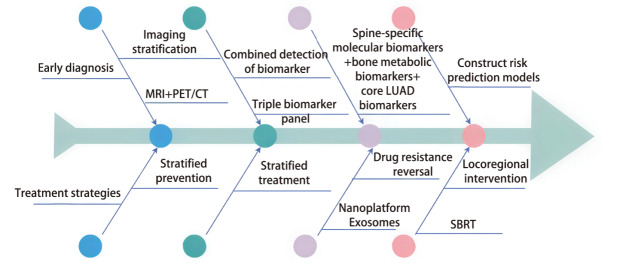
临床诊疗流程。构建LUAD脊柱转移早筛、标志物检测、风险预测与分层干预一体化诊疗体系。

## 5 总结与展望

LUAD脊柱转移是驱动基因异常、EMT激活及脊柱特殊微环境协同介导的定向转移过程，而非普通骨转移的简单延续。目前，多模态影像融合、循环肿瘤标志物检测、AI预测模型及靶向、耐药逆转等技术，为该病早期诊疗提供了技术支撑。但目前脊柱特异性分子调控机制尚不明确，相关研究循证医学证据不足，难以向临床转化。因此，未来研究需实现从“泛骨转移干预”到“脊柱定向干预”的转变，重点围绕多中心队列研究、特异性靶点临床转化、AI辅助诊疗落地等具体方向开展，增强研究临床实用价值。

在靶点方面，聚焦脊柱定向转移与治疗耐药的核心问题，解析肿瘤-脊柱微环境互作机制，筛选验证关键特异性靶点，明确其与患者转移进展、预后的相关性，验证靶向干预的安全性与有效性，优化靶向给药方案。

在临床研究方面，构建多中心、大样本、前瞻性LUAD脊柱转移专属临床队列，整合患者临床、影像、多组学及长期随访数据，搭建标准化临床数据库，验证诊断、疗效及预后标志物，明确不同治疗方案的适配人群，提升研究循证等级。

在AI辅助诊疗方面，依托多中心数据，优化LUAD脊柱微转移AI诊断模型，搭建分子联合影像无创早诊体系，提高微小病灶检出效率。同时运用AI完成病灶定位与放疗方案设计，结合Batson椎静脉解剖特点优化给药策略，解决脊柱特殊结构带来的诊疗精准度不足问题，实现AI技术与临床诊疗的深度融合，以有效破解临床早期诊断难、治疗耐药、疗效不佳等痛点，改善患者长期预后与生存质量。
